# Optimized Periocular Template Selection for Human Recognition

**DOI:** 10.1155/2013/481431

**Published:** 2013-07-31

**Authors:** Sambit Bakshi, Pankaj K. Sa, Banshidhar Majhi

**Affiliations:** Department of Computer Science and Engineering, National Institute of Technology Rourkela, Odisha 769008, India

## Abstract

A novel approach for selecting a rectangular template around periocular region optimally potential for human recognition is proposed. A comparatively larger template of periocular image than the optimal one can be slightly more potent for recognition, but the larger template heavily slows down the biometric system by making feature extraction computationally intensive and increasing the database size. A smaller template, on the contrary, cannot yield desirable recognition though the smaller template performs faster due to low computation for feature extraction. These two contradictory objectives (namely, (a) to minimize the size of periocular template and (b) to maximize the recognition through the template) are aimed to be optimized through the proposed research. This paper proposes four different approaches for dynamic optimal template selection from periocular region. The proposed methods are tested on publicly available unconstrained UBIRISv2 and FERET databases and satisfactory results have been achieved. Thus obtained template can be used for recognition of individuals in an organization and can be generalized to recognize every citizen of a nation.

## 1. Introduction

**Figure 1:**
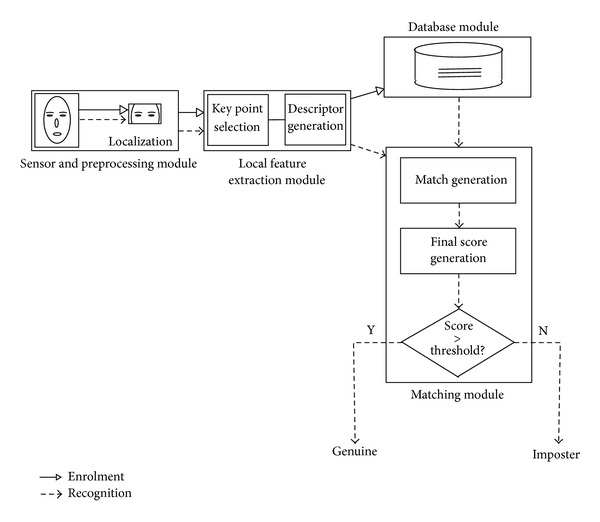
Working model of periocular biometric system.

A biometric system comprises a physical or behavioral trait of a person through which he or she can be recognized uniquely. Computer aided identification of a person through face biometric has grown its importance through the last decade and researchers have attempted to find unique facial nodal points. However, change of facial data with expression and age makes it challenging for recognition through face. A stringent necessity to identify a person on partial facial data has been felt in such scenario. There are forensic applications where antemortem information is a partial face. These motives led researchers to derive auxiliary biometric traits from facial image, namely, iris, ear, lip, and periocular region. Recognizing human through iris captured under near infrared (NIR) illumination and constrained scenario yields satisfactory recognition accuracy while recognition under visual spectrum (VS) and unconstrained scenario is relatively challenging. In particular, VS periocular image has been exploited to examine its uniqueness as there exists many nodal points. Classification and recognition through periocular region show significant accuracy, given the fact that periocular biometric uses only approximately 10% of a complete face data (illustrated in Section 4.1).  Figure 1 illustrates the working model of a biometric system that employs region around the eye (periocular region) as a trait for recognition. Face is one of the primitive means of human recognition.

**Figure 2:**
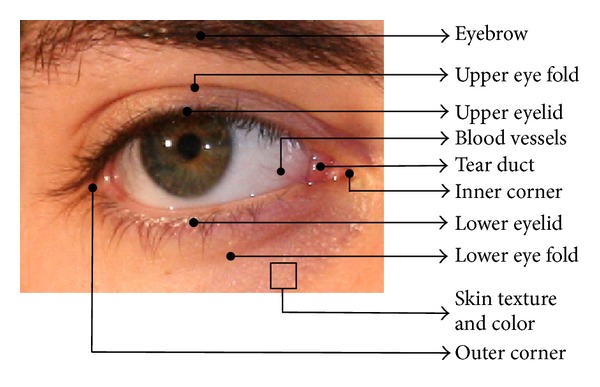
Important features from a periocular image.

Periocular (peripheral area of ocular) region refers to the immediate vicinity of the eye, including eyebrow and lower eye fold as depicted in Figure 2. Face recognition has been main attention of biometric researchers due to its ease of unconstrained acquisition and the uniqueness. Face is proven to have approximately 18 feature points [1] which can comprise in formation of a unique template for authentication. The major challenges in face detection faced by the researchers are due to change of human face with age, expression, and so forth. With the advent of low-cost hardware to fuse multiple biometrics in real time, the emphasis began to extract a subset of face which can partially resolve the aforementioned issues listed in Table 1. Hence the investigation towards ear, lip, and periocular has started gaining priority. Furthermore, capturing eye or face image automatically acquires periocular image. This gives the flexibility of recognizing an individual using the periocular data along with iris data without extra storage or acquisition cost. Moreover, periocular features can be used when an iris image does not contain subtle details, which mostly occurs due to poor image quality. Periocular biometric also comes into play as a candidate for fusion with face image for better recognition accuracy.

This paper approaches to fit an optimal boundary to the periocular region which is sufficient and necessary for recognition. Unlike other biometric traits, edge information is not the required criteria to exactly localize periocular region. Rather periocular region can be localized where the periphery of eye contains no further information. Researchers have considered a static rectangular boundary around the eye to recognize human and termed the localized rectangle as periocular region. However, this approach is naive as the same static boundary does not work for every face image (e.g., when the face image is captured through different distances from the camera, or when there is a tilt of face or camera during acquisition). So there is a need of deriving a dynamic boundary to describe periocular region. While deciding the periocular boundary, the objective of achieving the highest recognition accuracy also needs to be maintained. The paper specifies few metrics through which periocular region can be optimally localized in scale and rotation invariant manner.

The rest of the paper is organized as follows: Section 2 describes the landmark works in the direction of recognition and classification through periocular region and analyzes the need for optimizing the periocular region considered for recognition pointed in Section 3. In Section 4, four methods of template optimization are described and subsequently Section 5 records experimental results obtained to establish the proposed methods. Finally Section 6 concludes with describing the decided periocular template which is optimal for human recognition and marks its importance for recognition from a large database.

## 2. Literature Review

Investigations have been made by researchers in the direction of localizing iris from high quality constrained eye images captured in NIR illumination.  Table 2 summarizes the comparative study of accuracy obtained by few benchmark iris localization technique. The results conclude that high localization accuracy has been achieved for NIR iris images. Several global and local matching techniques have been applied for matching NIR iris images and researchers have got high accuracy. However, when it comes to recognize a person only through his iris image captured under visible spectrum, the results have been observed to be unsatisfactory. So researchers have been motivated to take into account not only iris but also its peripheral regions while recognizing visible spectrum images.

The task of recognition is more challenging than classification and hence draws more attention. The most commonly used feature extraction techniques in context of periocular recognition are Scale Invariant Feature Transform, Local Binary Pattern. Tables 3 and 4 outline the methods used and performance obtained towards periocular classification and recognition in visual spectrum images, respectively. However, the portion of eye on which it is applied is not computationally justified in the literature. Any arbitrary rectangular portion centering the eye has been taken into account without questioning the following.

- Will the accuracy obtained from this arbitrary boundary increase if a larger region is considered?
- How much of the considered periocular region is actually contributing to recognition?
- Is there any portion within this arbitrary considered periocular region which can be removed and still comparable accuracy can be achieved?

The derivation of optimal dynamic periocular region gives a simultaneous solution to the aforementioned questions.

## 3. Why Optimal Template for Periocular Region Is Required

Unlike other biometric traits, periocular region has no boundary defined by any edge information. Hence periocular region cannot be detected through differential change in pixel value in different directions. Rather the location of boundary is the region which is smooth in terms of pixel intensity, that is, a region with no information. The authors of [2] have localized the periocular region statically by taking a rectangle having dimension 6*R*_iris_ × 4*R*_iris_ centering the iris where *R*_iris_ defines the radius of the iris. But this localization method fails when the eye is tilted or gaze is not frontal. Moreover, the method presumes the location of iris center to be accurately detectable. However, iris center cannot be detected for some eye images due to low-resolution nature of the image.

The objective of the paper is to attain a dynamic boundary around the eye that defines periocular region. The region hence derived should have the following properties: (a) should be able to recognize humans uniquely, (b) should be achievable for low-quality VS images, (c) should contain main identifiable features of eye region identifiable by a human being, and (d) no subset of the derived periocular region should be equally potent as the derived region for recognition.

The optimally selected periocular template can be a template to hold identity of an individual. If such template can be generated for the whole nation, it can serve as authorized identity (i.e., biometric passport [23]) of every citizen of the nation.

## 4. Proposed Periocular Template Selection Methods

To achieve the above stated properties, four different dynamic models are proposed through which periocular region can be segmented out. These models are based on (a) human anthropometry, (b) demand of the accuracy of biometric system, (c) human expert judgement, and (d) subdivision approach.

### 4.1. Through Human Anthropometry

In a given face image, face can be extracted out by neural training to the system or by fast color-segmentation methods. The color-segmentation methods detect skin region in the image and find the connected components in such a region. Depending on connected components having skin color, the system labels the component largest in size as face.  Algorithm 1 proposes a binary component analysis based skin detection. The thresholds are experimentally fitted to obtain highest accuracy in segmenting skin region in face images comprising skin colors with different skin tones. The algorithm takes RGB face image as input. It first converts the face image to *YC*_*b*_ *C*_*r*_ color space and normalizes the pixel values. In the next step, the average luminance value is calculated by summing up the *Y* component values of each pixel and dividing the total number of pixels in the image. A brightness compensated image is generated depending on the value of average luminance as specified in the algorithm. In the obtained brightness compensated image, compound condition is applied and a thresholding is performed to obtain the skin-map finally. Through connected component analysis of the skin map in *YC*_*b*_ *C*_*r*_ color space, open eye region can be obtained as explained in Algorithm 2. The reason of segmenting open eye region is to obtain the nonskin region within detected face, which can be labeled as eye and thus to achieve approximate location of eye center.

Once the eye region is detected, the iris center can be obtained using conventional pupil detection and integrodifferential approach for finding the iris boundary and a static boundary can be fitted. As described earlier, the authors of [2] bounded periocular region with 6*R*_iris_ × 4*R*_iris_ rectangle centering the iris center. But no justification is produced in the paper regarding the empirically taken height and width of this periocular boundary. This process of finding periocular boundary has prerequisite of knowledge of coordinates of iris center and radius of iris.

Anthropometric analysis [24] of human face and eye region gives the information regarding the ratio of eye and iris and ratio of width of face and eye. A typical block diagram in Figure 6 depicts the ratios of different parts of human face with respect to height or width of face. From the analysis, it is found that

(1) widthperiocular=widtheyebrow=0.67×heightface2,heightperiocular=2×d(eyebrow,eyecenter)=2×(0.21+0.072)widthface2=0.49×widthface2,

where *d*_(eyebrow,eyecenter)_ denotes the distance between center of eyebrow and eye center:

(2) $$\begin{array}{c} \frac{{\text{height}}_{\text{eye}}}{{\text{width}}_{\text{eye}}}=\frac{0.49}{0.67}\times \frac{{\text{width}}_{\text{face}}/2}{{\text{height}}_{\text{face}}/2}=0.73\times \frac{{\text{width}}_{\text{face}}}{{\text{height}}_{\text{face}}}, \end{array}$$

(3) areaperiocular=widthperiocular×heightperiocular=0.67×heightface2×0.49widthface2=0.33×heightface2×widthface2=0.33π×(π×heightface2×widthface2)=0.11×areaface.

This information can be used to decide the boundary of periocular region. In (1), width and height of eye are expressed as a function of the height and width of human face. Hence to gauge the width and height of periocular template boundary, there is no need to have knowledge of iris radius. However, knowledge of coordinates of iris center is necessary. From these information, a bounding box can be fit composing all visible portions of periocular region, for example, eyebrow, eyelashes, tear duct, eye fold, eye corner, and so forth. This approach is crude and dependent on the human supervision or intelligent detection of these nodal points in human eye.

Further, from (2), it is observable that either information of the height or width of periocular region is sufficient to derive the other parameter, provided that the aspect ratio of face is known. This aspect of the localization of periocular is used in Section 4.2. Equation (3) considers elliptical model to represent face while finding the ratio of periocular region and area of a human face. It justifies the usefulness of using an optimally selected periocular template for human recognition rather than a full face recognition system.

This method achieves periocular localization without knowledge of iris radius. Hence it is suitable for localization of periocular region for unconstrained images where iris radius is not detectable by machines due to low-quality, partial closure of eye, or luminance of the visible spectrum eye image.

However, to make the system work in more unconstrained environment, periocular boundary can be achieved through sclera detection, for the scenario when iris cannot be properly located due to unconstrained acquisition of eye or when the image captured is a low-quality color face image captured from a distance.

#### 4.1.1. Detection of Sclera Region and Noise Removal

(1) The input RGB iris image  *im*  is converted to grayscale image *im_gray*.
(2) The input RGB iris image  *im*  is converted to HSI color model where *S* component of each pixel can be determined by
  (4) $$\begin{array}{c} S=1-\frac{3}{R+G+B}\left[\min\left(R,G,B\right)\right], \end{array}$$
  where *R, G, B *denotes the Red, Green, and Blue color component of a particular pixel. Let the image hence formed containing *S* component of each pixel is *saturation*_*im*.
(3) If *S* < *τ* where *τ* is a predefined threshold, then that pixel is marked as sclera region, else as a nonsclera region. Authors in [25] have experimented with *τ* = 0.21 to get a binary map of sclera region through binarization of *saturation*_*im* as follows: *sclera*_*noisy* = *saturation*_*im* < *τ*. Only a noisy binary map of sclera *sclera*_*noisy* can be found through this process, in which white pixels denote noisy sclera region and black pixels denote non-sclera region.
(4) *im_bin* is formed as follows: for every nonzero pixel (*i*, *j*) in *sclera*_*noisy*,
  (5) im_bin(i,j)=average  intensity  of  17×17  windowaround  the  pixel(i,j)in  im_gray
  for every zero pixel (*i*, *j*) in  *sclera*_*noisy*,
  (6) $$\begin{array}{c} im\_bin\left(i,j\right)=0. \end{array}$$
(5) *sc* *le* *ra*_*adaptive* is formed as follows:
  (7) $$\begin{array}{c} sclera\_adaptive\left(i,j\right)=\{\begin{array}{cc} 0, & \text{if}\;sclera\_noisy\left(i,j\right)=1\;\;\text{or}\\ & im\_gray\left(i,j\right)<im\_bin\left(i,j\right)\\ 1, & \text{otherwise}. \end{array} \end{array}$$
(6) All binary connected components present in *sclera*_*adaptive* are removed except the largest and second largest components.
(7) If size of the second largest connected component is less than 25% of that of the large one, it is interpreted that the largest component is the single sclera detected and the second largest connected component is removed hence. Else both components are retained as binary map of sclera.

After processing these above specified steps, the binary image would only contain one or two components describing the sclera region, after removing noises.

#### 4.1.2. Content Retrieval of Sclera Region

After a denoised binary map of sclera region within an eye image is obtained, it is necessary to retrieve the information about sclera, whether two parts of sclera on two sides of iris are separately visible, only one of them is detected, or both parts of sclera are detected as a single component.

**Figure 3:**
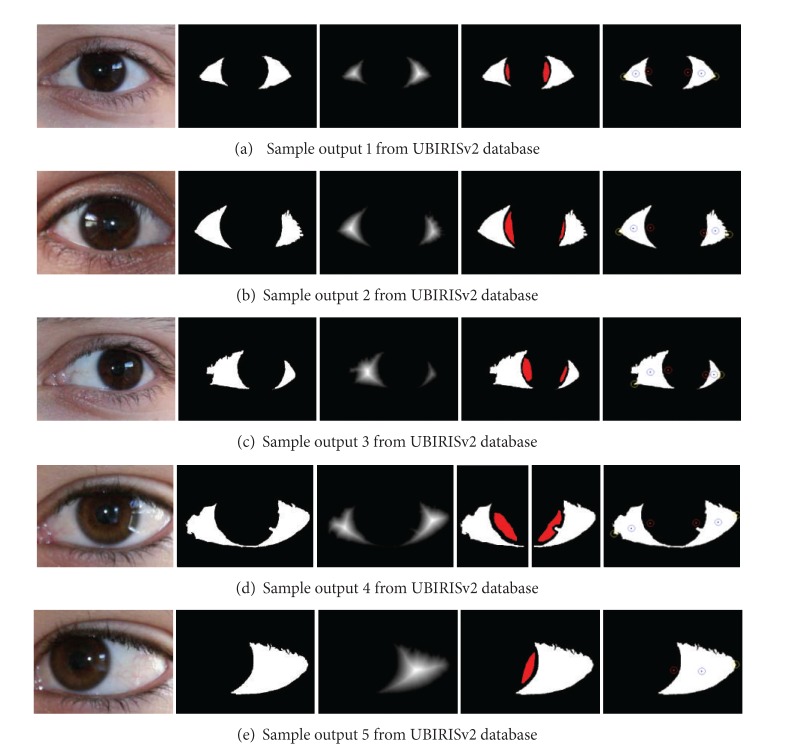
Result of nodal point detection through sclera segmentation.

There can be three exhaustive cases in the binary image found as sclera: (a) the two sides of the sclera is connected and found as a single connected component, (b) two sclera regions are found as two different connected components, and (c) only one side of the sclera is detected due to the pose of eye in the image. If the number of connected components is found to be two, then it is classified as aforementioned Case b (as shown in Figures 3(a), 3(b), and 3(c)) and two components are treated as two portions of sclera. Else, if a single connected component is obtained, it is checked for the ratio of length and breadth of the best fitted oriented bounding rectangle. If the ratio is greater than 1.25, then it belongs to aforementioned Case a, else belongs to Case c (shown in Figure 3(e)). For the aforementioned Case a, the region is subdivided into two components (through detecting minimal cut that divides the joined sclera into two parts) as shown in Figure 3(d) and further processing is performed.

#### 4.1.3. Nodal Points Extraction from Sclera Region

Each sclera is subjected to following processing through which three nodal points are detected from each sclera region, namely (a) center of sclera, (b) center of concave region of sclera, and (c) eye corner. So in general cases where two parts of the sclera are detected, six nodal points will be detected. The method of nodal point extraction is illustrated below.

1. *Finding Center of Sclera*. The sclera component is subjected to a distance transform where the value of each white pixel (indicating pixels belonging to sclera) is replaced by its minimum distance from any black pixel. The pixel which is farthest from all black pixels will have highest value after this transformation. That pixel is labeled as center of sclera.
2. *Finding Center of Concave Region of Sclera*. The midpoints of every straight line joining any two border pixels of the detected sclera component are found out as shown in Figure 5. The midpoints lying on the component itself (shown by red point between *P*_1_ and *P*_2_ in Figure 5) are neglected. The midpoints lying outside the component (shown by yellow point between *P*_3_ and *P*_4_ in Figure 5) are taken into account. Due to discrete computation of straight lines, midpoints of many straight lines drawn in aforementioned way overlap on a single pixel. A separate matrix having the same size as the sclera itself is introduced, which is having zero value of each pixel initially. For every valid midpoint, the value of corresponding pixel in this new matrix is incremented. Once this process is over, more than one connected components of nonzero values will be obtained in the matrix signifying concave regions. The largest connected component is retained while others are removed. The pixel having maximum value in the largest component is labeled as the center of concave region.
3. *Finding the Eye Corner*. The distances of all pixels lying on boundary of sclera region from the sclera center are also calculated to find the center of sclera as described above. The boundary pixel which is farthest from the center of the sclera is labeled as the eye corner.

The result of extracting these nodal points from eye image helps in finding the tilt of eye along with the position of iris in eye.  Figure 3 depicts five sample images from UBIRISv2 dataset and the outputs obtained from their processing through the aforementioned nodal point extraction technique. This information can be useful in localization of periocular region.

### 4.2. Through Demand of Accuracy of Biometric System

**Figure 4:**
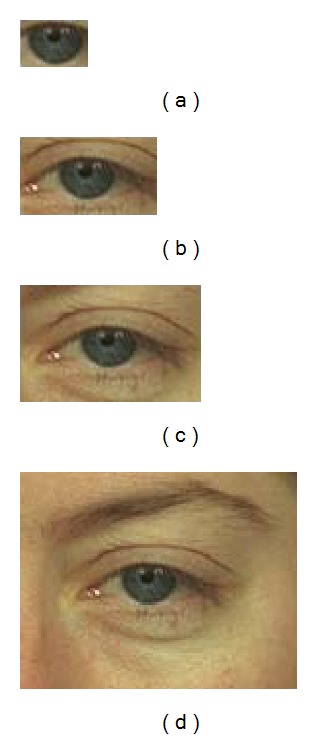
Cropped images from an iris image centering at pupil center.

**Figure 5:**
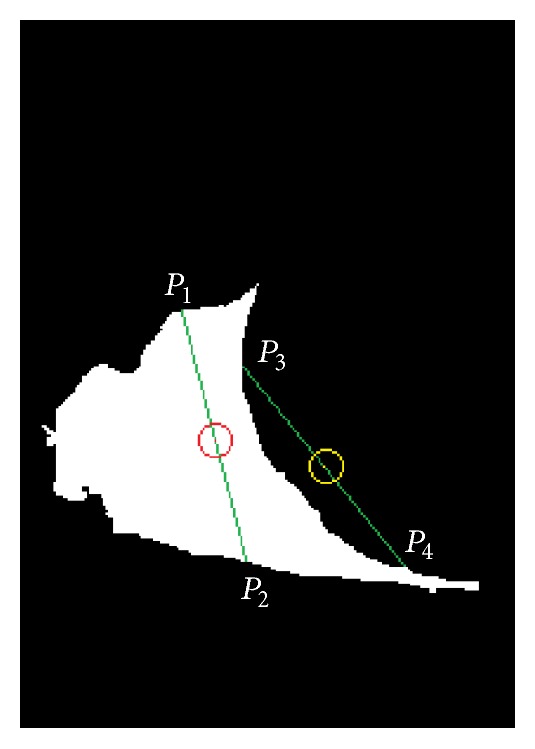
Method of formation of concave region of a binarized sclera component.

**Figure 6:**
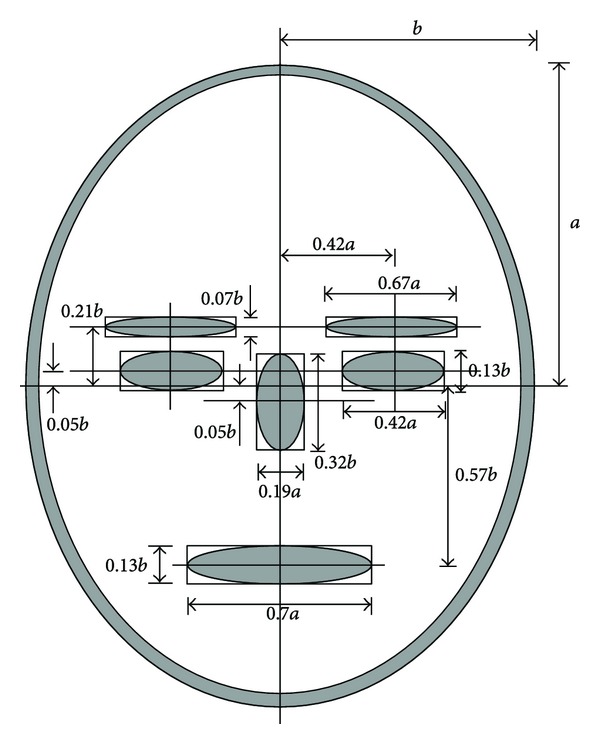
Different ratios of portions of face from human anthropometry.

Beginning with the center of the eye (pupil center), a bounding rectangular box is taken of which only encloses the iris.  Figure 4 shows how the eye images changes when it is cropped with pupil center and the bounding size is gradually increased. The corresponding accuracy of every cropped image is tested. In subsequent steps the coverage of this bounding box is increased with a width of 3% of the diameter of the iris and the change in accuracy is observed. After certain iterations of this procedure, the bounding box will come to a portion of periocular region where there is no more change in intensity; hence the region is low entropic. Hence no more local feature can be extracted from this region even if the bounding box is increased. In such scenario, the saturation accuracy is achieved, and on the basis of saturation accuracy, the corresponding minimum bounding box is considered as the desired periocular region. As the demand of different biometric systems may vary, the bounding box corresponding to certain predefined accuracy can also be segmented as periocular region. Similar results have also been observed for FERET database.

The exact method of obtaining the dynamic boundary is as follows.

1. For *i* = 0 to 100, follow the steps 2 to 4.
2. For each image in database, find approximate iris location in eye image.
3. For each image in database, centering at the iris center, crop a bounding box whose width *w* = 100 + 3 × *i*% of diameter of iris, height *h* = 73% of  *w*.
4. Find accuracy of the system with this image size.
5. Observe the change in accuracy with *w*.

**Figure 7:**
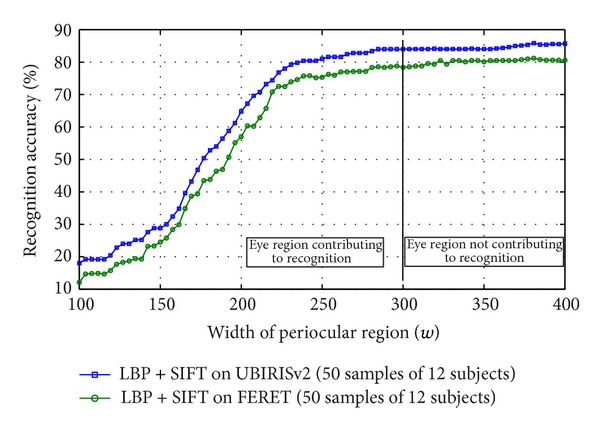
Change of accuracy of periocular recognition with change in size of periocular template tested on subset of UBIRISv2 and FERET datasets.

Figure 7 illustrates a plot of accuracy against *w* which shows that the accuracy of the biometric system saturates after a particular size of the bounding box. Increasing the box further does not increase the accuracy. To carry out this experiment, Local Binary Pattern (LBP) [26] along with Scale Invariant Feature Transform (SIFT) [27] are employed as feature extractor from the eye images. First, LBP is applied and resulting image is subjected for extracting local feature through SIFT. In the process, a maximum accuracy of 85.64% is achieved while testing with randomly chosen 50 eye images of 12 subjects from UBIRISv2 dataset [28]. When the same experiment is executed for randomly chosen 50 eye images of 12 subjects from FERET dataset [29], a maximum accuracy of 78.29% is achieved. These saturation accuracy values are obtained when a rectangular boundary of width 300% of diameter of iris is considered or a wider rectangular eye area is taken into consideration. To validate the experiment run on the sample strongly, the same experiment was conducted on complete UBIRISv2 and FERET dataset which yielded 85.43% and 78.01% accuracy, respectively. This concludes that a subset of a large database can be employed to find the optimal template size and the result found can be used on whole dataset for cropping of images. So to minimize template size without compromising in accuracy, the smallest wide rectangle with saturation accuracy can be used as localization boundary to periocular region. It is also observed that the region beyond 300% of diameter of iris, though does not participate in recognition, increases the matching time as shown in Figure 11. This is also another reason of removing the redundant eye region to make the recognition process fast.

**Figure 8:**
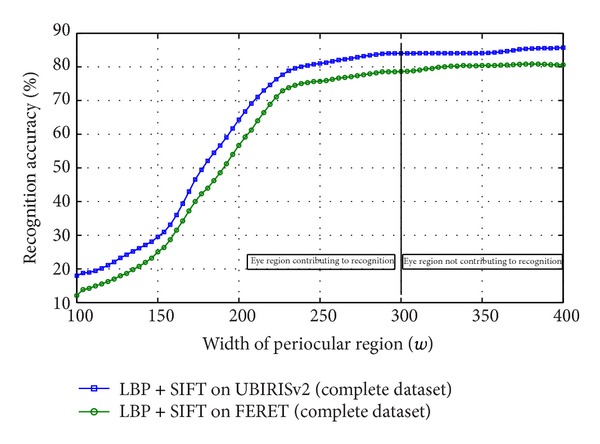
Change of accuracy of periocular recognition with change in size of periocular template tested on full UBIRISv2 and FERET datasets.

**Figure 9:**
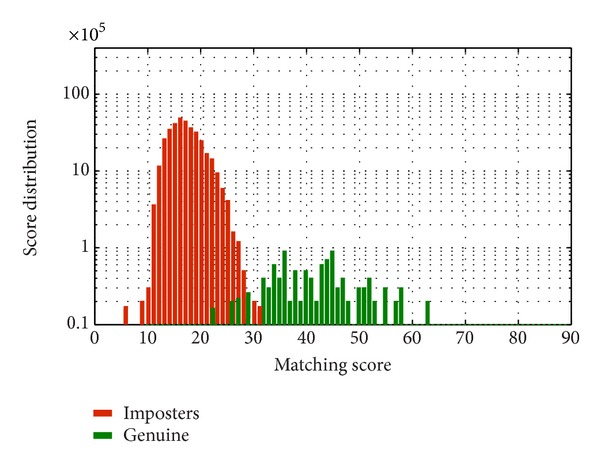
Distribution of scores for imposter and genuine matching tested on full UBIRISv2 dataset applying LBP + SIFT on periocular template having width as 300% of the iris diameter.

**Figure 10:**
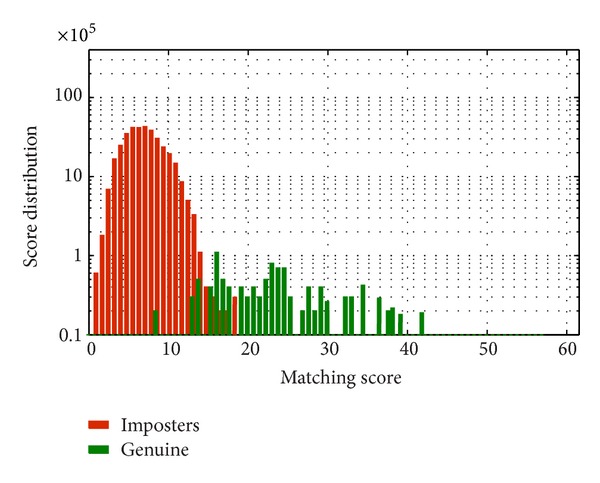
Distribution of scores for imposter and genuine matching tested on full FERET dataset applying LBP + SIFT on periocular template having width as 300% of the iris diameter.

**Figure 11:**
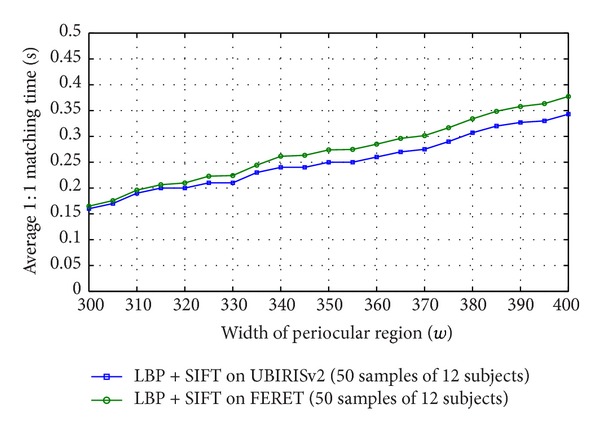
Change of 1 : 1 matching time with change in size of periocular template tested on full UBIRISv2 and FERET datasets.

To validate this experiment, the same experiment has been carried out once again on full database of UBIRISv2 and FERET. The obtained accuracy values as depicted in Figure 8 ensure the experimental objective that there is no significant feature in periocular region beyond 300% of diameter of iris which can contribute to recognition. The score distribution of imposter and genuine scores is shown in Figures 9 and 10.

### 4.3. Human Expert Judgement on Importance of Portions of Eye

Human expertise has been utilized to decide a sorted order of importance of different sections of periocular region towards recognition [17]. This information can be used to detect only the most important section in human eye that is most important towards recognition. If that section is not found in human eye region, the captured image is marked as Failure to Acquire (FTA) and not used for recognition. Hence a predecision on the quality of live query template can increase the accuracy of the system by reducing false rejections. However, this technique is human-supervised while enrolling an image in the database and while a live query comes. The human expert has to verify whether the most important portion of eye is visible in the image and has to guide the biometric system accordingly.

### 4.4. Through Subdivision Approach and Automation of Human Expertise

During enrolment phase of a biometric system, a human expert needs to verify manually whether the captured image includes expected region of interest. Through automated labeling different sections of an eye, it can be stated which portion of eye is necessary for identification (from human expert knowledge already discussed) and an automated FTA detection system can be made. Hence there is no need of a human expert for verifying the existence of important portions of human eye in an acquired eye image.

The challenge in incorporating this strategy in localization of periocular region is the automatic detection of portions of human eye like eyelid, eye corner, tear duct, lower-eyefold, and so forth. An attempt to do subdivision detection in eye region can be achieved through color detection and analysis and applying different transformations.

## 5. Experimental Results

There are four methods explained through which an optimal periocular template can be selected for biometric recognition. The first two methods explained in Sections 4.1 and 4.2 are experimentally evaluated using publicly available FERET and UBIRISv2 databases. A brief description of the two databases used for evaluation are illustrated in Table 5. A total of $\left(\begin{array}{c} 11102\\ 2 \end{array}\right)=61621651$ genuine and imposter matching among images from UBIRISv2 and $\left(\begin{array}{c} 14126\\ 2 \end{array}\right)=99764875$ genuine and imposter matching among images from FERET database are experimented to claim the proposition of optimality.

Anthropometry based approach performs accurately along with proper skin detection and sclera detection in eye region. The sample outputs are shown in Figure 3 which are found to be proper when evaluated against ground truth.

**Figure 12:**
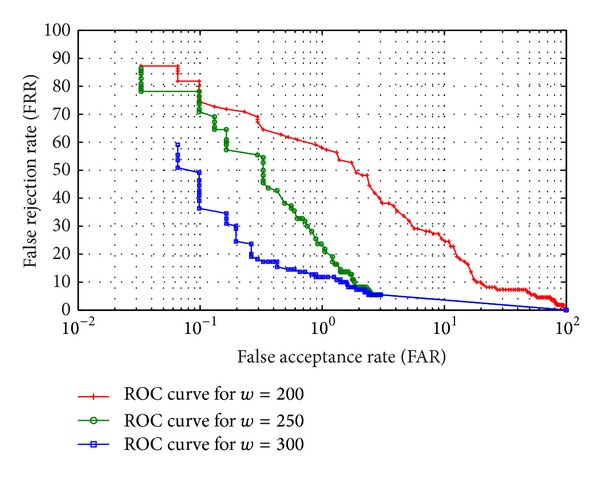
Receiver Operating Characteristic (ROC) curve for different template sizes of periocular region for UBIRISv2.

**Figure 13:**
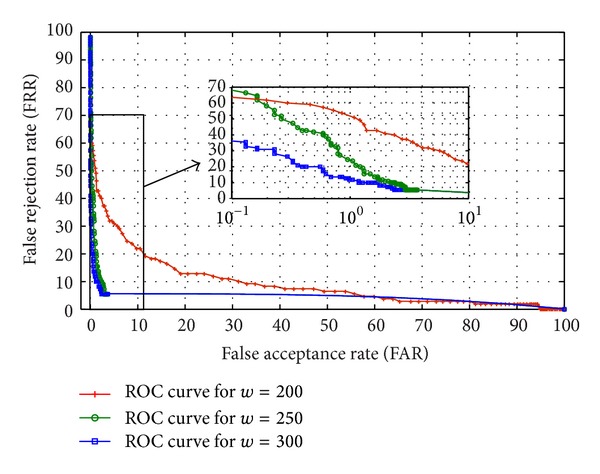
Receiver Operating Characteristic (ROC) curve for different template sizes of periocular region for FERET.

**Figure 14:**
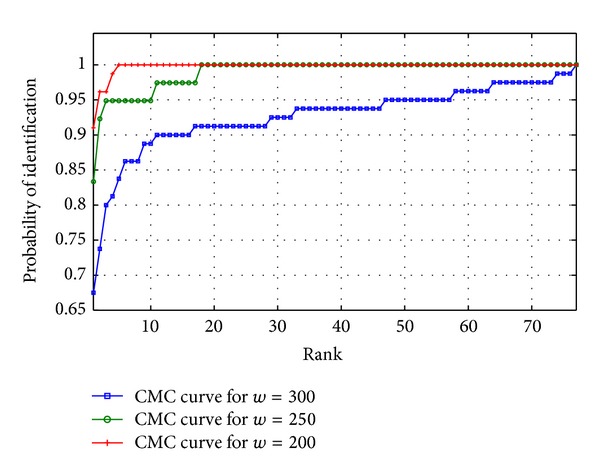
Cumulative Match Characteristic (CMC) curve for different template sizes of periocular region for UBIRISv2.

**Figure 15:**
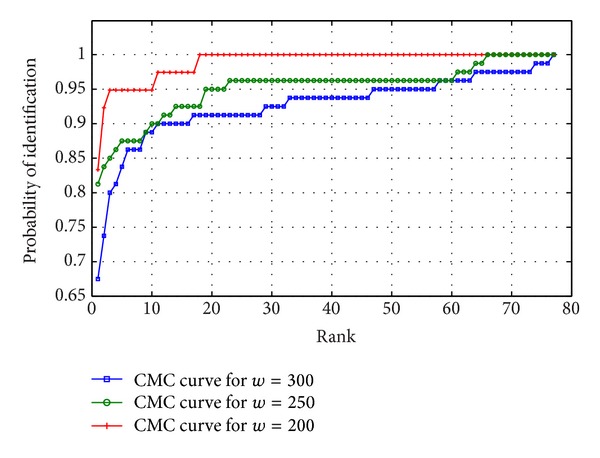
Cumulative Match Characteristic (CMC) curve for different template sizes of periocular region for FERET.

Saturation accuracy based approach performs with an accuracy more than 80% with noisy and low-resolution images of UBIRISv2 and FERET, which marks the efficiency of the proposed approach. To analyse the performance more deeply, Receiver Operating Characteristic (ROC) curve is experimented out when the width of the periocular region is 200%, 250%, and 300% of the diameter of iris region, respectively. ROC curve depicts the dependence of false rejection rate (FRR) with false acceptance rate (FAR) for change in the value of threshold. The curve is plotted using linear, logarithmic, or semilogarithmic scales. As plotted in Figures 12 and 13, it is obvious to conclude that the system performs better with low FAR when *w* = 300 than when *w* = 200 and 250. Hence the ROC curve reveals that the portions of eye lying between 200% and 300% of diameter of iris are very much responsible for the recognition and feature-dense part of a periocular image. Furthermore to have a 1 : *n*  matching analysis, Cumulative Match Characteristic (CMC) curves representing the probability of identification at various ranks are also experimented out when the width of the periocular region is 200%, 250%, and 300% of the iris region, respectively (shown in Figures 14 and 15). The *d*′ index [31] measures the separation between the arithmetic means of the genuine and imposter probability distribution in standard deviation units is defined as follows

(8) $$\begin{array}{c} d^{\prime }=\frac{\sqrt{2}\left|\mu_{\text{genuine}}-\mu_{\text{imposter}}\right|}{\sqrt{\sigma_{\text{genuine}}^{2}+\sigma_{\text{imposter}}^{2}}}, \end{array}$$

where *μ* and *σ* are mean and standard deviation of genuine and imposter scores.  Table 6 yields the change of *d*′ index of recognition when the width of periocular region is varied. The value of *d*′ increases monotonically from 1.23 to 2.85 for UBIRISv2 dataset and from 1.19 to 2.69 for FERET dataset with incremental change in *w*. An incremental nature in the values of *d*′ for *w* = 100 to 300 and an insignificant change in the value of *d*′ for *w* = 300 to 400 also establishes the existence of a boundary between regions contributing and not contributing to recognition.

Human expert judging is experimented by Hollingsworth et al. [17] and the results are used towards the direction of optimal periocular localization. Human subjects are asked which part of eye they feel to be the most important for recognition. Most of the subjects voted that blood vessels are the most important feature to recognize an individual from VS eye image. This information is used to infer which sub-portions of eye must belong to the optimal periocular region for it to be a candidate for recognition. Removal of those important regions will lead to rejection of the template.

Subdivision approach needs manual supervision in the process of proper labeling of the different portions of human eye. Once the enrolled templates are labeled by the expert, an optimal part of the template can be selected for recognition. The method is tested on FERET database and yielded proper localization of periocular region.

## 6. Conclusions

Recent research signifies why recognition through visual spectrum periocular image has gained so much importance and how the present approaches work. While developing recognition system for a large database, it is a crucial factor to optimize the template size. Existence of any redundant region in template will increase the matching time but will not contribute to increase the accuracy of matching. Hence removal of redundant region of the template should be accomplished before the matching procedure. As recognition time of identification is dependent on database size *n*, hence a decrease of 1 : 1 matching time of *t* will actually decrease* nt* matching time for identification in total. As *n* is large (in the range of 10^9^ practical cases), *nt* is a significant amount of time, especially when concurrent matching is implemented in distributed biometric systems. The paper prescribes four metrics for the optimization of visual spectrum periocular image and experimentally establishes their relevance in terms of satisfying expected recognition accuracy. These methods can be used to localize the periocular region dynamically so that an optimized region can be selected which is best suitable for recognition in terms of two contradictory objectives: (a) minimal template size, and (b) maximal recognition accuracy.

## Figures and Tables

**Algorithm 1:**
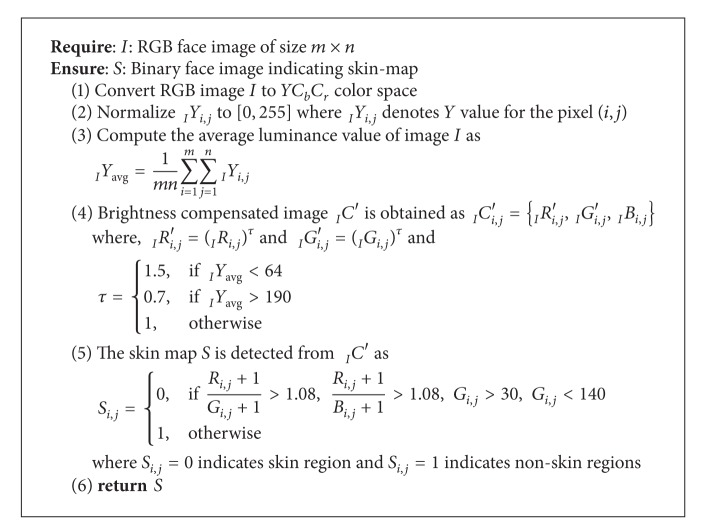
Skin_Detection.

**Algorithm 2:**
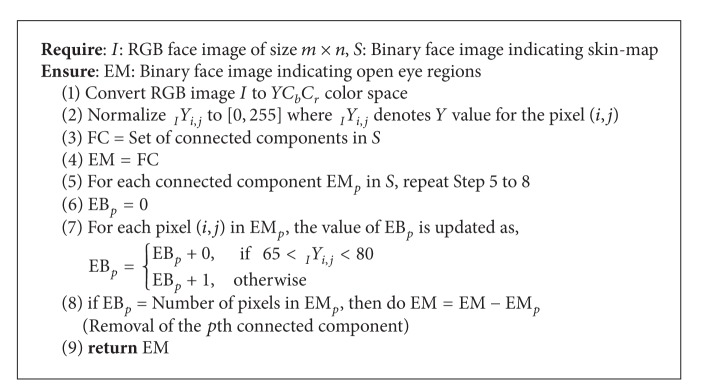
Open_Eye_Detection.

**Table 1:** Comparison of biometric traits present in human face.

Trait	Advantages	Possible challenges
Iris	High-dimensional feature can be extracted, difficult to spoof, permanence of iris, secured within eye folds, and can be captured in noninvasive way	Yields accuracy in NIR images than VS images, cost of NIR acquisition device is high, low recognition accuracy in unconstrained scenarios, low recognition accuracy for low resolution, occlusion due to use of lens, eye may close at the time of capture, do not work for keratoconus and keratitis patients

Face	Easy to acquire, yields accuracy in VS images, most available in criminal investigations	Not socially acceptable for some religions, full face image makes database large, variation with expression and age

Periocular	Can be captured with face/iris region without extra acquisition cost	Can be occluded by spectacle, less features in case of infants

Lip	Existence of both global and local features	Difficult to acquire, less acceptable socially, shape changes with human expression

Ear	Easy segmentation due to presence of contrast in the vicinity	Difficult to acquire and can be partially occluded by hair

**Table 2:** Performance comparison of some benchmark NIR iris localization approaches.

Year	Authors	Approach	Testing database	Accuracy results
2002	Camus and Wildes [3]	Multiresolution coarse-to-fine strategy	Constrained iris images (640 without glasses, 30 with glasses)	Overall 98% (99.5% for subjects without glasses and 66.6% for subjects wearing glasses)

2004	Sung et al. [4]	Bisection method, canny edge-map detector, and histogram equalization	3,176 images acquired through a CCD camera	100% inner boundary and 94.5% for collarette boundary

2004	Bonney et al. [5]	Least significant bit plane and standard deviations	108 images from CASIA v1 and 104 images from UNSA	Pupil detection 99.1% and limbic detection 66.5%

2005	Liu et al. [6]	Modification to Masek's segmentation algorithm	317 gallery and 4,249 probe images acquired using Iridian LG 2200 iris imaging system	97.08% rank-1 recognition

2006	Proença and Alexandre [7]	Moment functions dependent on fuzzy clustering	1,214 good quality, 663 noisy images from 241 subjects in two sessions	98.02% on good data set and 97.88% on noisy data set

2008	Pundlik et al. [8]	Markov random field and graph cut	WVU nonideal database	Pixel label error rate 5.9%

2009	He et al. [9]	Adaboost-cascade iris detector for iris center prediction	NIST Iris Challenge Evaluation (ICE) v 1.0, CASIA-Iris-V3-lamp, UBIRISv1.0	0.53% EER for ICEv1.0 and 0.75% EER for CASIA Iris-V3-lamp

2010	Liu et al. [10]	*K*-means cluster	CASIAv3 and UBIRISv2.0	1.9% false positive and 21.3% false negative (on a fresh data set not used to tune the system)

2010	Tan et al. [11]	Gray distribution features and gray projection	CASIAv1	99.14% accuracy (processing time 0.484 s/image)

2011	Bakshi et al. [12]	Image morphology and connected component analysis	CASIAv3	95.76% accuracy with processing (0.396 s/image)

**Table 3:** Survey on classification through periocular biometric.

Authors	Classification type	Algorithm	Classifier	Testing database	Accuracy (%)
Abiantun and Savvides [13]	Left versus right eye	Adaboost, Haar, Gabor features	LDA, SVM	ICE	89.95%
Bhat and Savvides [14]	Left versus right eye	ASM	SVM	ICE, LG	Left eye 91%, right eye 89%
Merkow et al. [15]	Gender	LBP	LDA, SVM, PCA	Downloaded from web	84.9%
Lyle et al. [16]	Gender and ethnicity	LBP	SVM	FRGC	Gender 93%, ethnic 91%

**Table 4:** Survey on recognition through periocular biometric.

Year	Authors	Algorithm	Features	Testing database	Performance results	
2010	Hollingsworth et al. [17]	Human analysis	Eye region	NIR images of 120 subjects	Accuracy of 92%	

2010	Woodard et al. [18]	LBP fused with iris matching	Skin	MBGC NIR images from 88 subjects	Left eye rank-1 recognition rate:	Iris 13.8% Periocular 92.5% Both 96.5%
					Right eye rank-1 recognition rate:	Iris 10.1% Periocular 88.7% Both 92.4%

2010	Miller et al. [19]	LBP	Color information, skin texture	FRGC neutral expression, different session	Rank-1 recognition rate:	Periocular 94.10% Face 94.38%
				FRGC alternate expression, same session	Rank-1 recognition rate:	Periocular 99.50% Face 99.75%
				FRGC alternate expression, a different session	Rank-1 recognition rate:	Periocular 94.90% Face 90.37%

2010	Miller et al. [20]	LBP, city block distance	Skin	FRGC VS images from 410 subjects	Rank-1 recognition rate:	Left eye 84.39% Right eye 83.90% Both eyes 89.76%
				FERET VS images from 54 subjects	Rank-1 recognition rate:	Left eye 72.22% Right eye 70.37% Both eyes 74.07%

2010	Adams et al. [21]	LBP, GE to select features	Skin	FRGC VS images from 410 subjects	Rank-1 recognition rate:	Left eye 86.85% Right eye 86.26% Both eyes 92.16%
				FERET VS images from 54 subjects	Rank-1 recognition rate:	Left eye 80.25% Right eye 80.80% Both eyes 85.06%

2011	Woodard et al. [22]	LBP, color histograms	Skin	FRGC neutral expression, a different session	Rank-1 recognition rate:	Left eye 87.1% Right eye 88.3% Both eyes 91.0%
				FRGC alternate expression, same session	Rank-1 recognition rate:	Left eye 96.8% Right eye 96.8% Both eyes 98.3%
				FRGC alternate expression, different session	Rank-1 recognition rate:	Left eye 87.1% Right eye 87.1% Both eyes 91.2%

**Table 5:** Detail of publicly available testing databases.

Database	Developer	Version	Number of images	Number of subjects	Resolution	Color model
UBIRIS	Soft Computing and Image Analysis (SOCIA) Group, Department of Computer Science, University of Beira Interior, Portugal	v1 [30] v2 [28]	1,877 11,102	241 261	800 × 600 400 × 300	RGB sRGB

FERET [29]	National Institute of Standards and Technology (NIST), Gaithersburg, Maryland	v4	14,126	1,191	768 × 512 384 × 256 192 × 128	RGB

**Table 6:** Change of *d*′ index with change of cropping of periocular region.

Width of periocular region (*w*)	100	150	200	250	300	350	400
Value of *d*′ index (for UBIRISv2 dataset)	1.23	1.60	2.05	2.34	2.61	2.72	2.85
Value of *d*′ index (for FERET dataset)	1.19	1.55	2.01	2.29	2.53	2.66	2.69

## Abbreviations

NIR: Near infrared
VS: Visual spectrum
LBP: Local Binary Pattern
SIFT: Scale Invariant Feature Transform
ROC: Receiver Operating Characteristic
CMC: Cumulative Match Characteristic
FTA: Failure to Acquire
FRR: False rejection rate
FAR: False acceptance rate.
